# Vaccinia virus protein C4 inhibits NF-κB activation and promotes virus virulence

**DOI:** 10.1099/vir.0.045070-0

**Published:** 2012-10

**Authors:** Stuart W. J. Ember, Hongwei Ren, Brian J. Ferguson, Geoffrey L. Smith

**Affiliations:** 1 Department of Pathology, University of Cambridge, Tennis Court Road, Cambridge CB2 1QP, UK; 2 Department of Virology, Faculty of Medicine, Imperial College London, Norfolk Place, London W2 1PG, UK

## Abstract

Vaccinia virus (VACV) strain Western Reserve protein C4 has been characterized and its function and contribution to virus virulence assessed. Bioinformatic analysis showed that C4 is conserved in six orthopoxvirus species and shares 43 % amino acid identity with VACV protein C16, a known virulence factor. A recombinant VACV expressing a C-terminally tagged version of C4 showed that, like C16, this 37 kDa protein is expressed early during infection and localizes to both the cytoplasm and the nucleus. Functional assays using a firefly luciferase reporter plasmid under the control of a nuclear factor kappa B (NF-κB)-dependent promoter demonstrated that C4 inhibits NF-κB activation at, or downstream of, the inhibitor of kappa kinase (IKK) complex. Consistent with this, C4 inhibited interleukin-1β-induced translocation of p65 into the nucleus. A VACV lacking the *C4L* gene (vΔC4) showed no significant differences from wild-type virus in growth kinetics or spread in cell culture, but had reduced virulence in a murine intranasal model of infection. vΔC4-infected mice exhibited fewer symptoms, lost less weight and recovered 7 days earlier than animals infected with control viruses expressing C4. Furthermore, bronchoalveolar lavage fluid from vΔC4-infected mice had increased cell numbers at day 5 post-infection, which correlated with reduced lung virus titres from this time onward. C4 represents the ninth VACV protein to inhibit NF-κB activation and remarkably, in every case examined, loss of each protein individually caused an alteration in virus virulence, despite the presence of other NF-κB inhibitors.

## Introduction

*Vaccinia virus* (VACV) is the prototypical member of the genus *Orthopoxvirus* (OPV) of the *Poxviridae*, a family of large, complex viruses with dsDNA genomes of 135 kb or more (Moss, 2007). Like other poxviruses, VACV replicates in cytoplasmic factories and encodes many proteins needed for virus transcription and DNA replication (Moss, 2007). VACV is the live vaccine used to immunize against smallpox, an eradicated human disease caused by the antigenically related OPV variola virus (Fenner *et al.*, 1988). After smallpox eradication, interest in VACV continued due to the development of recombinant VACVs as candidate vaccines for other micro-organisms, for instance hepatitis B virus (Smith *et al.*, 1983; Moss *et al.*, 1984; Paoletti *et al.*, 1984), and because VACV is a useful expression vector (Moss, 1996), a tool for immunologists (Bennink *et al.*, 1984, 1986: Yewdell *et al.*, 1985; McMichael *et al.*, 1986) and a good model system for studying virus–host interactions (Cudmore *et al.*, 1995; Alcamí & Smith, 1996; Doceul *et al.*, 2010).

The VACV genome contains approximately 200 ORFs (Goebel *et al.*, 1990) with a highly conserved central region encoding proteins required for VACV transcription, replication and assembly (Upton *et al.*, 2003; Gubser *et al.*, 2004). In contrast, the more variable terminal regions encode proteins that are non-essential for virus replication in cell culture, but which affect virus host range, virulence and immunomodulation *in vivo*. The latter group includes proteins that are secreted from infected cells to bind cytokines, chemokines, interferons (IFNs) or complement factors, and intracellular proteins that inhibit apoptosis, synthesize steroid hormones or block signalling cascades leading to activation of transcription factors that promote expression of IFNs and pro-inflammatory molecules (Smith, 1994; Alcami, 2003; Seet *et al.*, 2003).

A subset of these VACV immunomodulatory proteins inhibit activation of nuclear factor kappa B (NF-κB), a transcription factor that is retained in the cytosol of resting cells bound to the inhibitor of kappa B alpha (IκBα). When upstream signalling pathways are activated by, for instance, engagement of the interleukin (IL)-1 receptor with IL-1β, tumour necrosis factor (TNF) receptor with TNF-α, or Toll-like receptors (TLRs) with their respective ligands, IκBα is phosphorylated, ubiquitinated and degraded. Consequently, NF-κB is released and translocates to the nucleus, where it activates NF-κB-responsive genes (Hayden & Ghosh, 2008). NF-κB is an important transcription factor for the expression of many pro-inflammatory molecules and therefore it is not surprising that viruses encode proteins that inhibit activation of NF-κB. VACV is no exception and encodes many proteins that target this pathway, including A46, A52, N1, B14, M2, K1, K7 and E3.

Protein A52 binds TNF receptor-associated factor 6 (TRAF6) and IL-1 receptor-associated kinase 2 (IRAK2) and inhibits NF-κB activation downstream of TLRs and the IL-1 receptor (Bowie *et al.*, 2000; Harte *et al.*, 2003). Protein A46 targets the host Toll–IL-1 receptor (TIR) adaptors myeloid differentiation factor 88 (MyD88), MyD88 adaptor-like, TIR domain-containing adaptor inducing IFN-β (TRIF) and the TRIF-related adaptor molecule, and thereby interferes with NF-κB activation (Bowie *et al.*, 2000; Stack *et al.*, 2005). Protein B14 binds to the inhibitor of kappa B kinase (IKK)β and thereby inhibits its activation and the consequential phosphorylation of IκBα (Chen *et al.*, 2006, 2008). Protein N1 was reported to bind the IKK complex (DiPerna *et al.*, 2004), but this has been disputed (Chen *et al.*, 2008) and so the mechanism by which N1 inhibits NF-κB activation remains uncertain. Protein M2 reduced ERK2 phosphorylation induced by phorbol myristate acetate and prevented p65 nuclear translocation (Gedey *et al.*, 2006). Proteins K7 and K1 inhibit NF-κB activation either by inhibiting TLR-induced signalling (K7) (Schröder *et al.*, 2008) or by preventing IκBα degradation (K1) (Shisler & Jin, 2004). Finally, protein E3 inhibits NF-κB activity in both PKR-dependent and -independent manners (Myskiw *et al.*, 2009), and also by antagonizing the RNA polymerase III–dsDNA-sensing pathway (Valentine & Smith, 2010).

This study reports a characterization of VACV strain Western Reserve (WR) protein C4, encoded by gene *C4L*. Protein C4 has a predicted size of 37.2 kDa and is conserved within other VACV strains and OPVs (http://www.poxvirus.org), suggesting an important function. Protein C4 also shows 43 % amino acid identity to VACV protein C16, which is an immunomodulator and affects virus virulence (Fahy *et al.*, 2008). However, C4 and C16 are functionally distinct because viruses lacking C16 exhibit an attenuated phenotype despite the presence of C4. Data presented here show that C4 is expressed early, is present in both the cytoplasm and the nucleus and inhibits activation of NF-κB at, or downstream of, the IKK complex. Despite being the ninth VACV protein assigned this function, deletion of C4 from the VACV genome caused attenuation of virus virulence. Possible explanations for this observation are discussed.

## Results

### Computational analysis of the *C4L* gene

VACV WR gene *C4L* (GenBank accession no. YP_232906) encodes a 37.2 kDa protein without a transmembrane domain or signal peptide (http://www.poxvirus.org) and without obvious cellular orthologues. The C-terminal sequence VTKYYI is very similar to VTKFYF present in the same position of the IL-1 receptor antagonist (IL1-ra) protein. This peptide is also conserved in the related VACV protein C16 (VTRFYF) (Fahy *et al.*, 2008) and peptides containing this sequence were reported to have immunosuppressive activity (Kluczyk *et al.*, 2002). C4 is conserved in seven of 15 VACV strains, including WR, Copenhagen, Lister (and derivatives such as LC16m0), which all contain the C-terminal VTKYYI peptide, but is absent from VACV strains chorioallantois vaccinia virus Ankara (CVA), modified vaccinia Ankara (MVA) and its derivatives, Acambis 2000 and its derivatives, 3737 and DUKE. C4 is also present in six of eight OPV species (95–99 % amino acid identity), including VACV, cowpox virus, camelpox virus (CMLV), taterapoxvirus (TATV), variola virus and monkeypox virus (MPXV), but not in ectromelia virus (ECTV) or horsepox virus. C16 is conserved (97–100 % amino acid identity) in all sequenced strains of VACV, but only in five OPV species (Fahy *et al.*, 2008). Notably, the three OPVs lacking C16 (CMLV, TATV and MPXV) encode C4. Outside OPVs, C4 showed limited conservation (23–32 % amino acid identity) in eight chordopoxviruses, including mule deer poxvirus, sheeppox virus and lumpy skin disease virus.

### Construction of *C4L* deletion, revertant and TAP VACVs

Several recombinant VACVs (strain WR) were constructed (Methods) to study the C4 protein within VACV-infected cells. These included a plaque-purified wild-type virus (vC4), a virus lacking the *C4L* gene (vΔC4) and a revertant virus in which the *C4L* gene was reinserted at its natural locus (vC4-Rev). To characterize the C4 protein during VACV infection in the absence of a C4 antibody, a virus expressing C4 from its natural promoter and TAP-tagged at the C terminus (vC4-TAP) was constructed. PCR utilizing *C4L* primers confirmed the presence of *C4L* in vC4, vC4-Rev and vC4-TAP and its absence in vΔC4 (Fig. S1, available in JGV Online). Analysis of genomic DNA by restriction endonuclease digestion showed that the only discernible difference between these viruses was at the *C4L* locus (data not shown).

### Analysis of C4 expression during VACV infection

**Fig. 1.**
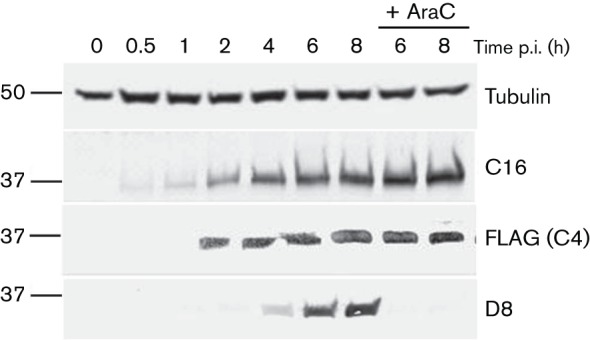
C4 is expressed early during infection. BSC-1 cells were infected with vC4-TAP at 10 p.f.u. per cell in the presence or absence of 40 µg AraC ml^−1^. At the indicated times p.i., the cells were washed with PBS and lysed. Samples were resolved by SDS-PAGE and analysed by immunoblotting with antibodies against the indicated proteins. The positions of molecular mass markers are indicated (kDa).

To determine when C4 is expressed, BSC-1 cells were infected with vC4-TAP in the presence or absence of cytosine arabinoside (AraC), an inhibitor of viral DNA replication and late protein expression, and extracts of cells were analysed by immunoblotting at different times post-infection (p.i.) (Fig. 1). C4–TAP was detected as a 37 kDa protein, consistent with its predicted size. Like protein C16 (Fahy *et al.*, 2008), the C4 protein was detected from 2 h p.i. onwards and in the presence of AraC, indicating early expression. In contrast, AraC inhibited expression of D8, a virion structural protein expressed late during infection (Niles & Seto, 1988). Immunoblotting with anti-α-tubulin confirmed equivalent loading of samples.

**Fig. 2.**
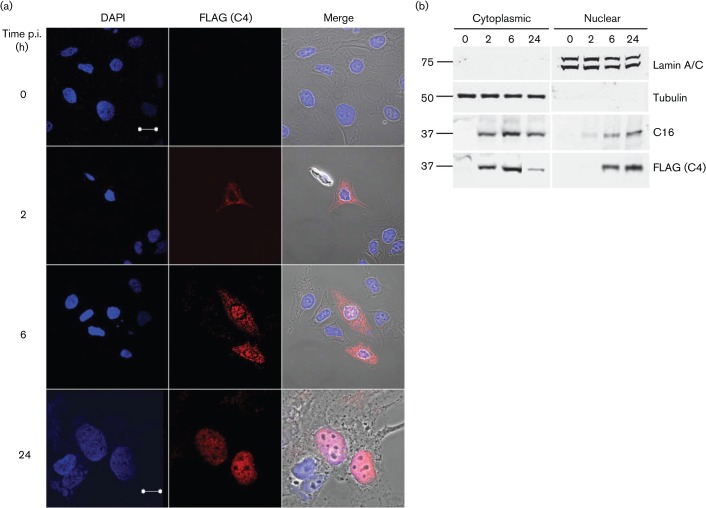
Subcellular localization of C4. (a) Immunofluorescence. HeLa cells were infected with vC4-TAP at 0.5 p.f.u. per cell for the indicated times. Cells were washed with PBS, fixed and stained with anti-FLAG mAb. The localization of C4/FLAG (red; middle panels), DNA stained with DAPI (blue; left panels) and phase-contrast/merged images (right panels) are shown. Bars, 20 µm (0–6 h); 10 µm (24 h). (b) Immunoblotting. BSC-1 cells were infected at 10 p.f.u. per cell with vC4-TAP for the indicated times, harvested and fractionated into cytoplasmic and nuclear fractions. Protein fractions were resolved by SDS-PAGE and analysed by immunoblotting with antibodies against the indicated proteins. The positions of molecular mass markers are shown (kDa).

The subcellular localization of C4 was investigated by infecting HeLa cells with vC4-TAP and then either examining fixed cells by immunofluorescence (Fig. 2a) or performing immunoblotting of biochemically fractionated cellular lysates (Fig. 2b). Consistent with temporal expression data, immunofluorescence detected the C4 protein from 2 h p.i. in the cytoplasm, but C4 subsequently became increasingly nuclear. By 6 h p.i., C4 was detectable within both cytoplasm and nucleus, but by 24 h it was predominantly nuclear. Immunoblotting of fractionated cells corroborated data obtained by immunofluorescence and showed that C16 had a distribution similar to that of C4 (Fig. 2b). Successful separation of cytoplasmic and nuclear compartments was confirmed by the presence of α-tubulin and lamin A/C in only the cytoplasmic and nuclear fractions, respectively.

### C4 does not affect virus replication in cell culture

**Fig. 3.**
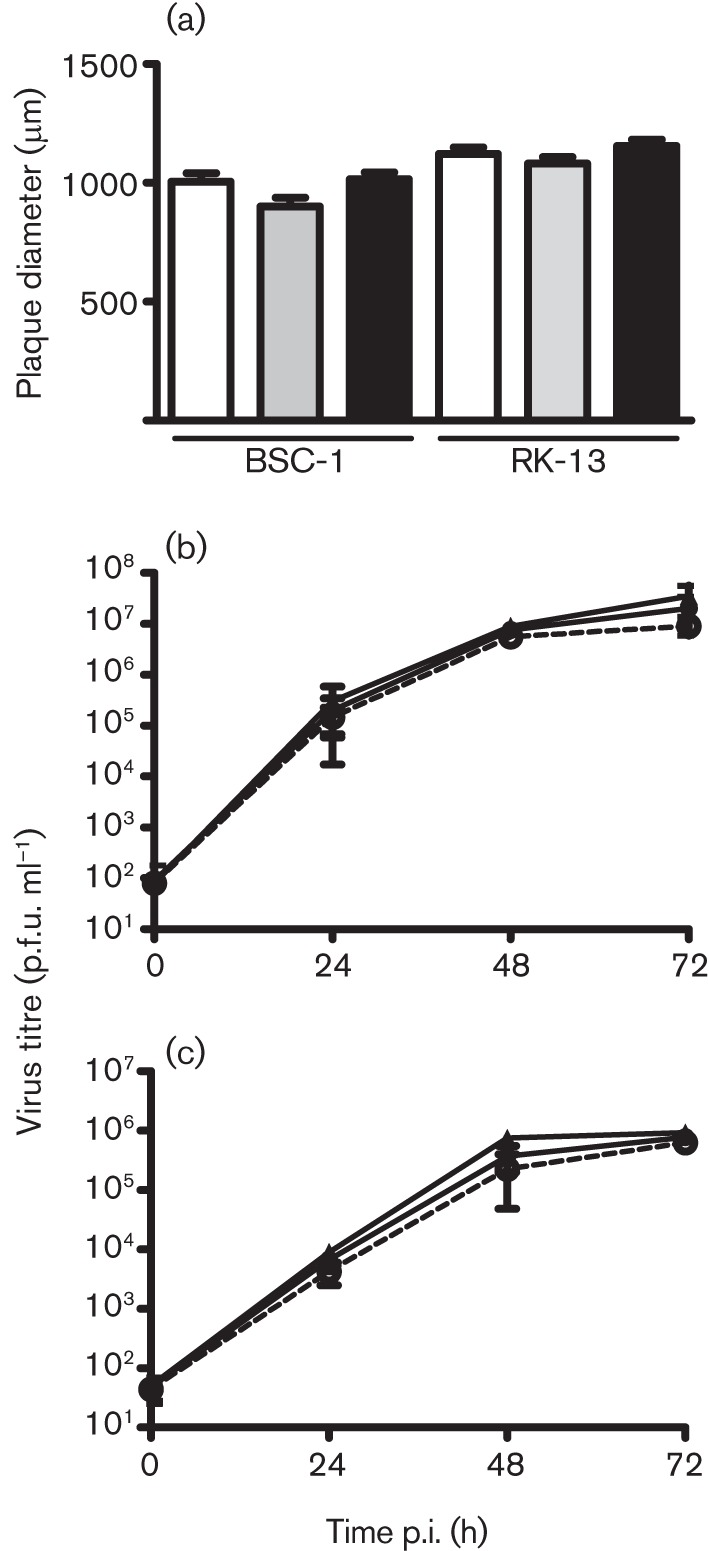
C4 is non-essential for virus replication and spread. (a) Plaque size. Monolayers of BSC-1 or RK-13 cells were infected with viruses (empty bars, vC4; shaded bars, vΔC4; filled bars, vC4-Rev) for 72 h. The sizes of 30 plaques were measured for each virus. Data are expressed as the mean±sd plaque diameter (μm). (b, c) Growth curves. BSC-1 cells were infected at 0.01 p.f.u. per cell and (b) intracellular and (c) extracellular virus were collected at the indicated times and titrated by plaque assay on BSC-1 cells. •, vC4; ○, vΔC4; ▴, vC4-Rev. Data are presented as the mean±sd log_10_(p.f.u.).

The fact that not all VACV strains contain gene *C4L* indicated that it was non-essential for virus replication, and this was confirmed for VACV strain WR by the isolation of the C4 deletion mutant, vΔC4. To ascertain whether C4 affected virus replication or spread, the size of plaque formed by vΔC4 was compared with that of control viruses in RK-13 and BSC-1 cells; no significant differences were observed (Fig. 3a). Next, the replication of vΔC4 in BSC-1 cells was investigated after infection at low (0.01) or high (10) m.o.i. and viruses in the intra- and extracellular fractions were titrated by plaque assay. Again, no differences were observed between vΔC4 and control viruses (Figs 3b, c and S2). Collectively, these data indicate that C4 is non-essential for virus replication and spread.

### C4 inhibits NF-κB activation

**Fig. 4.**
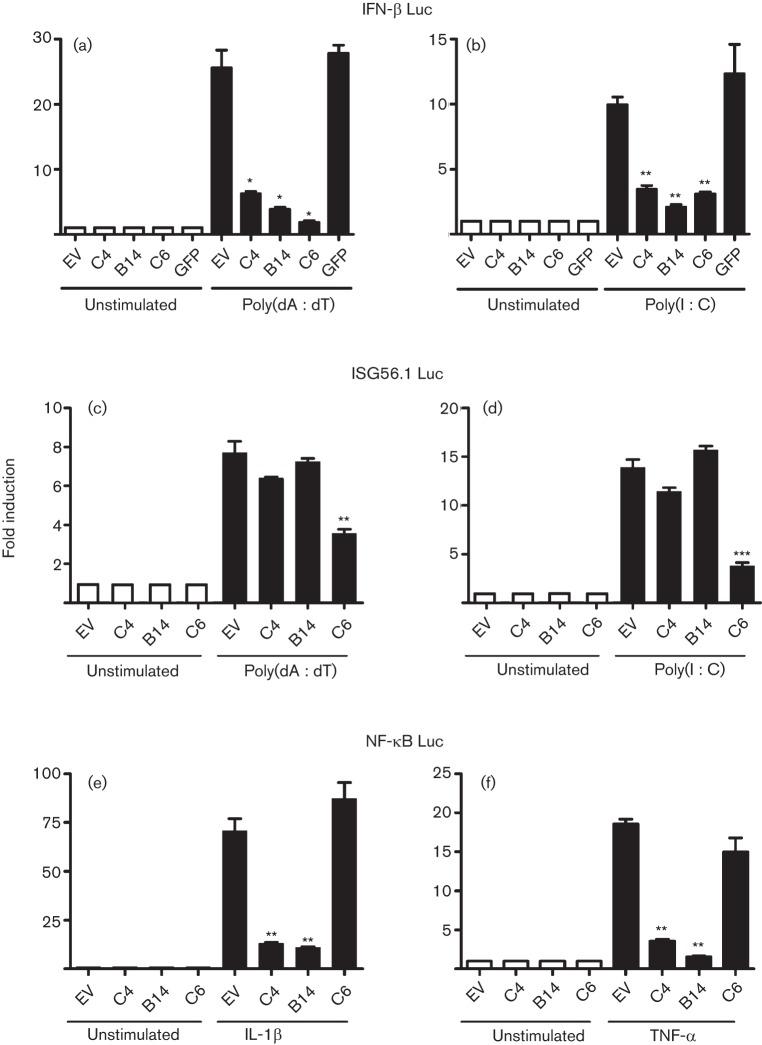
C4 inhibits NF-κB activation. HEK293T cells were transfected with a firefly luciferase reporter plasmid under the control of an (a, b) IFN-β-, (c, d) ISG56.1- or (e, f) NF-κB-dependent promoter, a *Renilla* luciferase transfection control, and a C4, B14, C6 or GFP expression plasmid or empty vector control (EV). Cells were (a–d) transfected 24 h later with (a, c) poly(dA : dT) or (b, d) poly(I : C) at 800 ng per well for 24 h or (e, f) stimulated directly with (e) IL-1β or (f) TNF-α at 40 ng ml^−1^ for 8 h and then harvested. Firefly luciferase activity was normalized to *Renilla* luciferase activity. Data are from one experiment representative of at least three, each performed in triplicate and presented as means±sd. *P<0.05; **P<0.005; ***P<0.0005, compared with EV.

Given that C4 was intracellular, its proposed possible function as an extracellular IL-1ra-like protein seemed improbable. Therefore, we investigated whether C4 inhibited intracellular signalling pathways, using a reporter plasmid with the IFN-β promoter driving expression of firefly luciferase. This was transfected into HEK293T cells that were stimulated subsequently by transfection with poly(dA : dT), a ligand for intracellular DNA sensors, or poly(I : C), a ligand of retinoic acid-inducible gene (RIG)-I-like receptors. These stimuli each induced luciferase activity, which was inhibited by C4 but not by a GFP control (Fig. 4a, b). Inhibition was also achieved by VACV protein B14, which inhibits NF-κB activation (Chen *et al.*, 2008), and by C6, which inhibits interferon regulatory factor (IRF) 3 activation (Unterholzner *et al.*, 2011), because the IFN-β promoter contains binding sites for both IRF3 and NF-κB.

To dissect the mechanism of action of C4, its ability to inhibit activation of IRF3, NF-κB and activator protein 1 (AP-1) was investigated with reporter plasmids specific for these transcription factors. To measure IRF3 activity, an ISG56.1-dependent promoter was stimulated by the transfection of poly(dA : dT) and poly(I : C). ISG56.1 promoter activity was inhibited by C6, a recently characterized antagonist of IRF3 activation (Unterholzner *et al.*, 2011), but not by C4 or B14 (Fig. 4c, d). Next, the effect of C4 on NF-κB-specific promoter activity was assessed using an NF-κB-dependent promoter (Fig. 4e, f). Stimulation of HEK293T cells with IL-1β and TNF-α resulted in a 70- and 18-fold induction of NF-κB promoter activity, respectively, and both were inhibited by C4. As expected B14, but not C6, was able to inhibit NF-κB activation (Chen *et a*l., 2008; Unterholzner *et al.*, 2011). Satisfactory stimulation of an AP-1 reporter plasmid was not achieved under several conditions tried and so the effect of C4 on this transcription factor could not be determined. Collectively, these data indicate that C4 inhibits IFN-β promoter activity by inhibiting NF-κB, but not IRF3, activation.

**Fig. 5.**
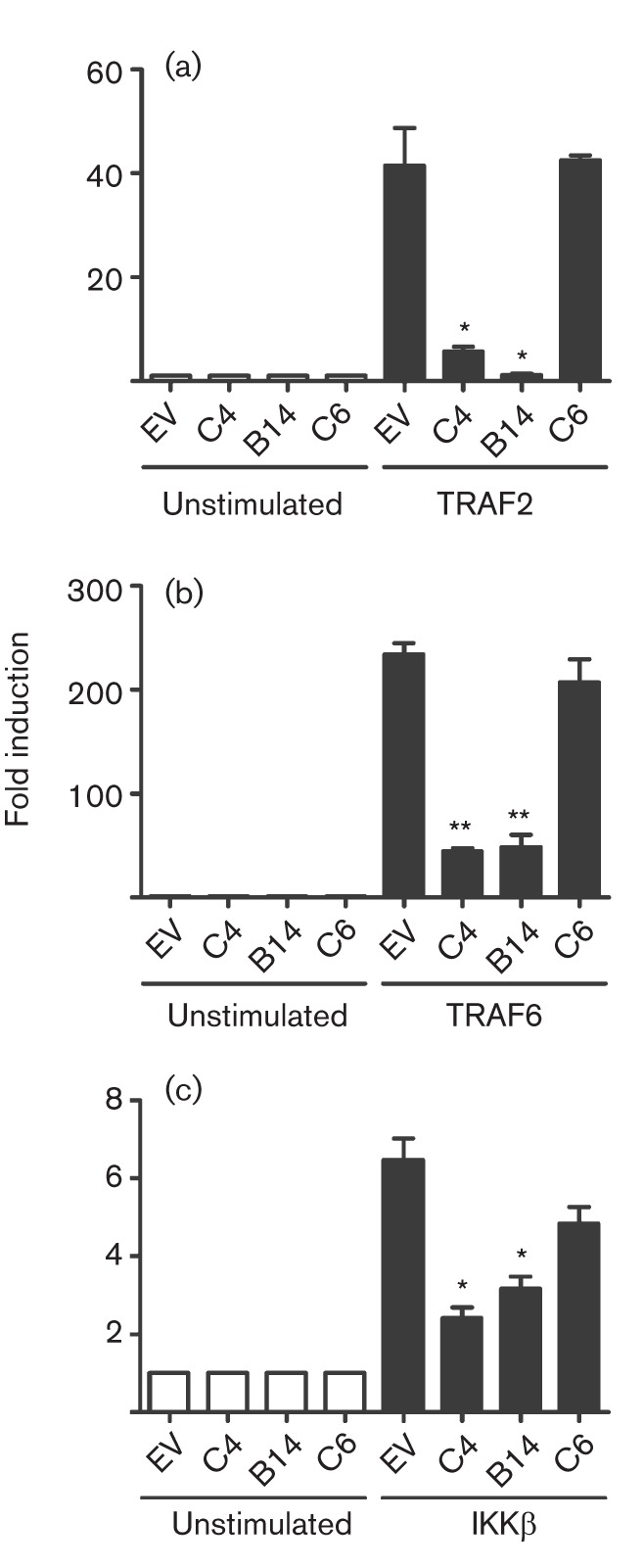
C4 inhibits NF-κB activation at or downstream of IKKβ. HEK293T cells were transfected with a firefly luciferase reporter plasmid under the control of an NF-κB-dependent promoter and (a) TRAF2, (b) TRAF6 or (c) IKK-β expression plasmids. Cells were co-transfected with a *Renilla* luciferase control and a C4, B14 or C6 expression plasmid or empty vector control (EV) for 24 h. Firefly luciferase activity was normalized to *Renilla* luciferase activity. Data are from one experiment representative of at least three, each performed in triplicate and presented as mean±sd. *P<0.05; **P<0.005, compared with EV.

**Fig. 6.**
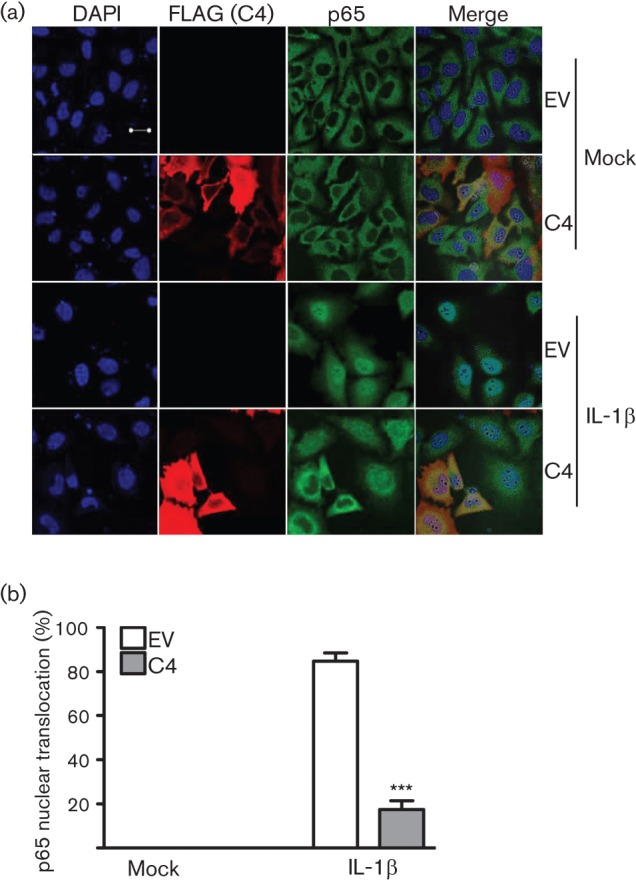
C4 blocks p65 translocation. (a) Immunofluorescence. HEK293T cells were transfected with a C4 expression plasmid or empty vector control (EV). After 24 h, cells were mock-stimulated or stimulated with 20 ng IL-1β ml^−1^ for 20 min. Cells were washed with PBS, fixed and stained with anti-FLAG (red) and anti-p65 (green) mAbs. DNA was stained with DAPI (blue) and phase-contrast/merged images (right panels) are shown. Bar, 20 µm. (b) Scoring: 90 cells per condition were quantified for nuclear translocation of p65. Data shown are from one experiment representative of three. ***P<0.0005, compared with EV.

The mechanism by which C4 inhibited NF-κB activation was investigated by overexpression of proteins acting at different stages in the signalling cascade. Expression of TRAF2, which acts downstream of the TNF receptor, or TRAF6, which acts downstream of the IL-1 receptor, both stimulated NF-κB expression and both pathways were inhibited by C4 and B14 (Fig. 5a, b). C4 was therefore inhibiting both IL-1- and TNF-mediated signalling and so was likely to act at, or downstream of, the position where these pathways converge. To test this hypothesis, NF-κB was activated by overexpression of IKKβ, part of the IKK complex (Fig. 5c); once again both C4 and B14, but not C6, inhibited this activation. Taken together, these data demonstrate that C4 inhibits the activation of NF-κB at, or downstream of, the IKK complex. This was confirmed by measuring the translocation of p65 into the nucleus in response to IL-1β stimulation (Fig. 6). In resting cells p65 was cytoplasmic but, in response to IL-1β, it became nuclear and this was inhibited by C4 expression (Fig. 6a, b).

### C4 affects virus virulence and cell recruitment

**Fig. 7.**
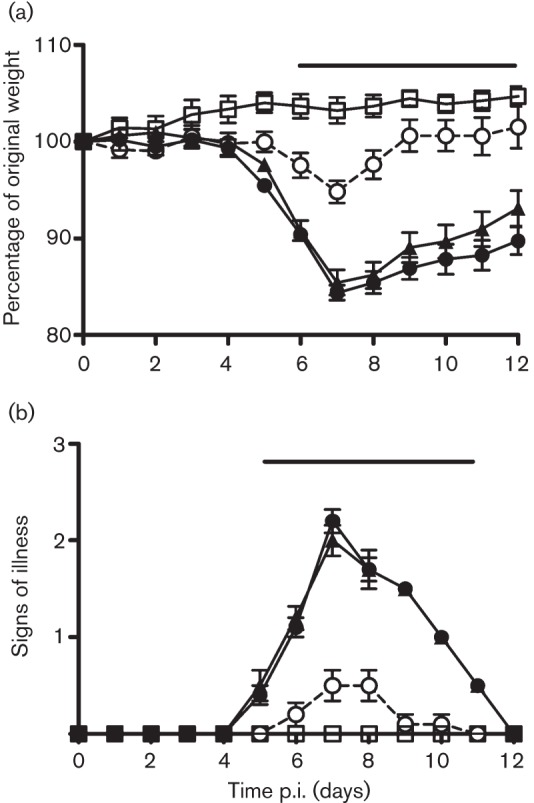
C4 is a virulence factor. BALB/c mice (*n* = 5) were infected intranasally with the indicated viruses and their weights and signs of illness were measured daily (Methods). (a) Weights. Data (means±sd) are expressed as a percentage of the mean weight of the same group of animals on day 0. (b) Signs of illness. The mean ±sem score of each group of animals is shown. □, Mock; •, vC4; ○, vΔC4; ▴, vC4-Rev. The horizontal bars indicate days on which the weight loss or signs of illness induced by vΔC4 was statistically different (*P*<0.05) from both vC4 and vC4-Rev.

**Fig. 8.**
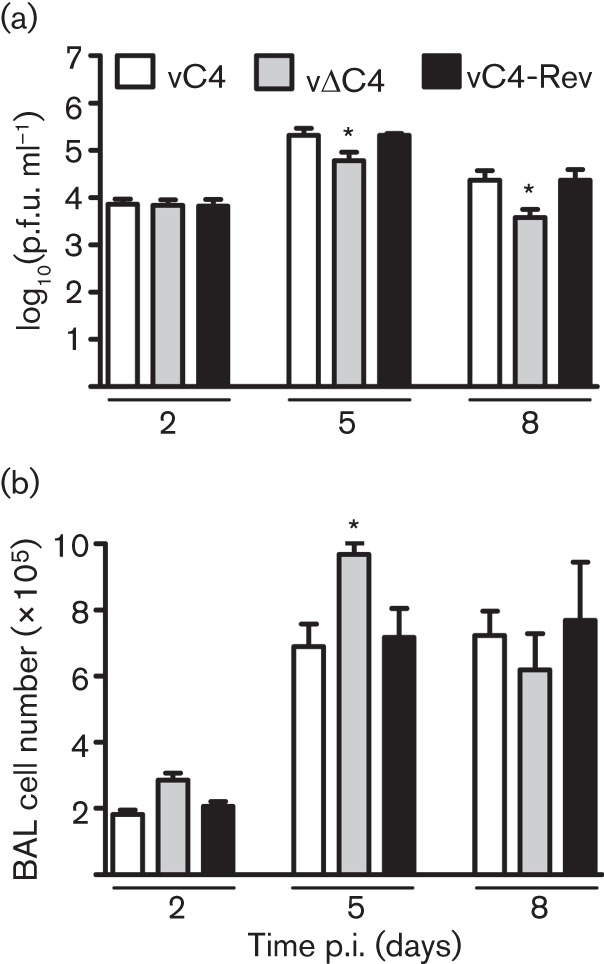
Lower virus titres and increased cell infiltration are associated with vΔC4 infection. BALB/c mice (*n* = 5) were infected intranasally with the indicated viruses. (a) Virus titres in lungs. At the indicated times, groups (*n* = 5) were sacrificed, lungs were removed and infectious virus (ml lung homogenate)^−1^ was determined by plaque assay. Data are presented as mean±sd titre. (b) Total number of viable cells in BAL fluid. At the indicated times, groups (*n* = 5) were sacrificed and cells were extracted from BAL fluid. Data are presented as mean±sd cell counts. *P<0.05 for vΔC4, compared with both vC4 and vC4Rev.

As C4 inhibited NF-κB activation, it was possible that C4 might affect virus virulence, and this was tested utilizing intradermal and intranasal murine models of VACV infection (Williamson *et al.*, 1990; Tscharke & Smith, 1999). In the intradermal model, there was no difference in lesion size in mice infected with vΔC4 compared with control viruses (Fig. S3). However in the intranasal model, mice infected with vΔC4 lost less weight, exhibited fewer signs of illness and recovered faster than mice infected with control viruses (Fig. 7a, b). These differences were statistically significant (*P*<0.05) from days 6 and 5 p.i., respectively. Measurement of virus titres in lungs at day 2 p.i. showed that all viruses had replicated to equivalent levels, demonstrating that, as *in vitro*, the loss of C4 did not impair virus replication *in vivo*. However, by days 5 and 8 p.i., vΔC4-infected mice had significantly lower virus titres than controls, suggesting more rapid clearance of virus in the absence of C4 (Fig. 8a). To investigate the basis for this phenotype, the number of viable cells in bronchoalveolar lavage (BAL) fluids was investigated on days 2, 5 and 8 p.i. (Fig. 8b). This revealed that, as early as day 2 p.i., there was an increase in the number of cells in BALs from vΔC4-infected mice, although this difference was not statistically significant. However, by day 5 a larger and statistically significant difference was seen. By day 8 p.i., there were fewer cells in the BAL fluid of mice infected with vΔC4 compared with controls. The recruitment of cells into BALs therefore showed quantitative and kinetic differences following infection by vΔC4, with earlier recruitment and higher numbers of cells, and more rapid resolution. Note that, at day 8 p.i., the number of cells present in BALs of mice infected with control viruses was equivalent to that at day 5, whereas in vΔC4-infected animals, the cell number had already declined from that at day 5, indicating recovery from infection. The more rapid and greater recruitment of cells into BAL fluids of vΔC4-infected mice correlated well with the reduced virus titres at days 5 and 8 p.i. C4 is therefore both an immunomodulator and a virulence factor.

## Discussion

Characterization of the C4 protein from VACV strain WR is reported. Data presented show that C4 is an intracellular, 37 kDa protein that is expressed early after infection and is non-essential for virus replication. Functional assays demonstrated C4 to inhibit NF-κB activation, acting at or downstream of the IKK complex, and C4 was shown to be a virulence factor in a murine intranasal model of infection.

The *C4L* gene is conserved (95–99 % amino acid identity) in six of eight OPV species and seven of 15 sequenced VACV strains. It is also related closely to VACV protein C16 and a family of OPV proteins (Fahy *et al.*, 2008). It was noteworthy that C4 is encoded by the three OPVs that do not encode C16 (CMLV, TATV and MPXV). The close relatedness of C4 and C16 (43 % amino acid identity) and the fact that C4 is present in those OPVs lacking C16 suggest that these proteins have an important role in the virus life cycle and may have partially overlapping functions. However, it is clear that C4 and C16 are not redundant because removal of either gene causes an alteration in virus virulence, as shown in this study and by Fahy *et al.* (2008).

The detection of C4 early during infection is consistent with the results of transcriptional analysis which reported that C4 mRNA was detectable within 30 min p.i. (Assarsson *et al.*, 2008). The expression of C4 also parallels that of C16, which is also made early during infection (Fahy *et al.*, 2008), and this timing is consistent with the immunomodulatory function of these proteins. VACV proteins that inhibit the innate immune response to infection are generally expressed rapidly after infection to prevent activation of signalling cascades leading to production of IFNs and pro-inflammatory cytokines and chemokines. One exception to this principle is the case of the VACV IL-1β-binding protein encoded by VACV strain WR gene *B15R*, which is expressed late during infection and controls the body temperature of infected animals (Smith & Chan, 1991; Alcamí & Smith, 1992, 1996).

The localization of C4 during infection is unusual and shows a predominant cytoplasmic expression early (2 h) p.i., but this changes with time and by 6 h it is in both nuclear and cytoplasmic fractions, and by 24 h is predominantly nuclear (Fig. 2). Similarly, the related protein C16 is also predominantly cytoplasmic early after infection and accumulates in the nucleus as infection progresses (Fahy *et al.*, 2008). C4 does not contain an identifiable nuclear-localization sequence, suggesting that it might bind to another viral or cellular protein to mediate this translocation, or that it can move between cytoplasmic and nuclear compartments by passive diffusion (which is possible for proteins ≤50 kDa). However, the strong nuclear predominance later during infection argues against simple diffusion for this localization. Proteins C6 and E3 are other examples of VACV immunomodulatory proteins that have a nucleocytoplasmic localization during the virus life cycle (Yuwen *et al.*, 1993; Unterholzner *et al.*, 2011).

The absence of C4 in several VACV strains and in ECTV indicated that the protein was non-essential for OPV replication and this was consistent with isolation of a VACV strain WR deletion mutant vΔC4. Despite this, a minor reduction in plaque size was noted, which, although not statistically significant with the sample size studied, was observed consistently in multiple cell types. Similarly, vΔC16 also produced a smaller plaque, but in that instance, the difference was large enough to be statistically significant (Fahy *et al.*, 2008). In the future, it will be interesting to investigate the plaque size of a virus lacking both genes. An analysis of virus replication kinetics *in vitro* and *in vivo* showed no difference between vΔC4 and control viruses.

Functional screens revealed C4 to be an inhibitor of IFN-β promoter activation in response to poly(dA : dT) and poly(I : C). Mechanistically, this was attributable to inhibition of activation of NF-κB rather than IRF3. C4 is therefore distinct from VACV protein C6, which inhibits IRF3 activation by binding to the adaptor proteins TANK, SINTBAD and NAP1 (Unterholzner *et al.*, 2011), but similar to B14, which inhibits NF-κB activation by binding to IKKβ (Chen *et al.*, 2008; Graham *et al.*, 2008; Benfield *et al.*, 2011). The position in the pathway leading to NF-κB activation at which C4 functions was deduced to be at, or downstream of, the IKK complex, because C4 inhibited activation mediated by overexpression of TRAF2, TRAF6 and IKKβ. This was consistent with its ability to block NF-κB activation by both IL-1β and TNF-α. C4 is therefore comparable to B14, which binds to IKKβ and prevents the phosphorylation of the IKKβ activation loop (Chen *et al.*, 2008; McCoy *et al.*, 2010; Benfield *et al.*, 2011).

Given the similar function of C4 and B14 and the fact that C4 represents the ninth VACV protein to inhibit NF-κB (see Introduction), it was surprising that loss of C4 caused an attenuated phenotype (Fig. 7). Furthermore, in all cases where the loss of an individual NF-κB inhibitor has been studied *in vivo* – A46 (Stack *et al.*, 2005), A52 (Harte *et al.*, 2003), B14 (Chen *et al.*, 2006), N1 (Bartlett *et al.*, 2002) and C4 (this paper) – there is also an attenuated phenotype, despite the presence of other inhibitors. These observations indicate that these proteins are non-redundant and that this non-redundancy might be explained in different ways. First, the proteins may have multiple functions, as has been demonstrated for N1, which is an inhibitor of both apoptosis and NF-κB activation (DiPerna *et al.*, 2004; Cooray *et al.*, 2007; Maluquer de Motes *et al.*, 2011). Second, the position in the NF-κB activation pathway at which an inhibitor functions could affect outcome. For instance, if an inhibitor blocked only IL-1β or TNF-α-induced NF-κB activation, the influence *in vivo* could be different from that of an inhibitor that acts on both pathways. Furthermore, crosstalk between NF-κB and other signalling pathways could also allow an inhibitor to influence outcomes *in vivo* differently, depending on where it acts.

A further surprise is that losses of C4 or B14 give different *in vivo* phenotypes, despite blocking the same pathway. With vΔB14, the attenuation was evident in the intradermal but not the intranasal model (Chen *et al.*, 2006), whereas with vΔC4, the opposite is observed. C4 represents another example where a phenotype is seen in only one model and illustrates the value of using both models, as noted previously (Tscharke *et al.*, 2002). Mechanistically, a propensity for an immunomodulator to show a phenotype in only one *in vivo* model has been attributed to its mechanism of action and the different inflammatory responses elicited by infection via either route (Tscharke *et al.*, 2002; Reading & Smith, 2003). In this case, given that B14 and C4 target the same pathway and at a similar position, the different *in vivo* phenotype is more surprising and suggests, perhaps, that one or both of these proteins have additional functions.

In summary, VACV protein C4 is an intracellular inhibitor of NF-κB and promotes virus virulence. C4 represents the ninth VACV inhibitor of NF-κB, and the fact that VACV has evolved so many inhibitors of this pathway highlights the importance of NF-κB signalling for the host response to virus infection. A notable feature of these inhibitors is that, in all cases where the virulence of viruses lacking individual proteins has been studied, reduced virulence is observed, despite the presence of the other inhibitors. This indicates additional complexity of these proteins and signalling pathways that is worthy of further investigation and may enhance our understanding of virus pathogenesis and innate immunity.

## Methods

### 

#### Cell culture.

BSC-1, CV-1 and HEK293T cells were grown in Dulbecco’s modified Eagle’s medium (Gibco) supplemented with 10 % FBS (Harlan Laboratories) that was heated at 56 °C for 1 h to inactivate complement, and penicillin/streptomycin (50 µg ml^−1^; Gibco). HeLa and RK-13 cells were grown in minimum essential medium (Gibco) supplemented as above and with the addition of non-essential amino acids (Sigma). All cell lines were maintained at 37 °C in a 5 % CO_2_ atmosphere.

#### Construction of plasmids.

For construction of a VACV lacking the *C4L* gene, 300–350 bp flanking regions of the *C4L* gene were amplified by overlapping PCR from VACV WR genomic DNA, and then cloned into the Z11 mammalian expression vector containing *Escherichia coli* guanine phosphoribosyltransferase (*Ecogpt*) and enhanced green fluorescent protein (*EGFP*) genes, generating plasmid Z11ΔC4, as described previously for another VACV gene (Unterholzner *et al.*, 2011). The 5′ DNA fragment was generated with oligonucleotides 5′-GCCACGCGTTCCAATTATCTTTACCG-3′ (L-FA) containing an *Mlu*I restriction site (underlined) and 5′-*CCCGGGTCCGGAGAGCTC*TAGTATTGGTTAAAAATGAAAATGG-3′ containing nucleotides from the 3′ fragment (italics) at the 5′ end. The 3′ DNA fragment was generated with oligonucleotides 5′-*GAGCTCTCCGGACCCGGG*TTTATATCACTACGG-3′ containing complementary sequence to the 5′ fragment (italics) and 5′-TAGCGGCCGCGGTGTCCTGTGTTCAGG-3′ (L-FB) containing a *Not*I restriction site (underlined). Left and right amplicons were joined by PCR using the L-FA and L-FB oligonucleotides to generate an FA-FB fragment. For construction of a C4 revertant virus in which the *C4L* gene was reinserted into its natural locus within the C4 deletion mutant, *C4L* together with the *C4L* flanking regions was amplified from VACV WR genomic DNA using oligonucleotides L-FA and L-FB and then cloned into Z11, generating plasmid Z11C4Rev.

The sequence of *C4L* was codon-optimized for expression in mammalian cells (GeneArt, Invitrogen Life Technologies). *C4L* was then subcloned into the mammalian expression vector pcDNA4.0/TO (Invitrogen) containing a C-terminal tandem affinity purification (TAP) tag consisting of two FLAG and two StrepII epitopes (Gloeckner *et al.*, 2007), generating plasmid C4-TAP. For construction of the TAP-tagged C4 revertant virus (vC4-TAPRev), *C4L* and the *C4L* left flanking region were cloned into the pcDNA4.0/TO vector and the *C4L* right flanking region was then cloned downstream of the TAP tag. The complete *C4L* locus and TAP tag was subsequently subcloned into the pUC13 mammalian expression vector modified to contain the *Ecogpt* and *EGFP* genes (Maluquer de Motes *et al.*, 2011), generating the plasmid pUC13-C4TAPRev. The fidelity of all plasmids constructed using PCR was confirmed by DNA sequencing.

#### Construction of recombinant VACVs.

C4 recombinant viruses were constructed by transfecting plasmid Z11ΔC4 into VACV-WR-infected CV-1 cells (to make vΔC4) or transfecting Z11C4Rev or pUC13-C4TAPRev into vΔC4-infected cells (to make vC4-Rev and vC4-TAPRev, respectively). Recombinant VACVs were subsequently isolated by transient dominant selection (Falkner & Moss, 1990) as described for the construction of other VACV deletion mutants (Chen *et al.*, 2006; Fahy *et al.*, 2008), using three cycles of plaque purification. The genotype of viruses was determined by *C4L* locus-specific PCR, and a plaque-purified wild-type virus (vC4) and a *C4L* deletion mutant virus (vΔC4) were identified and amplified. A revertant virus expressing C4 and a recombinant virus expressing C4 with a C-terminal TAP tag were isolated from vΔC4 by the same methodology. The genomes of all viruses were confirmed to have the predicted structure by PCR and restriction endonuclease digestion of genomic DNA.

#### Immunoblotting.

BSC-1 cells were infected with the indicated viruses and cell lysates prepared as described previously (Bartlett *et al.*, 2002). Antibodies were from the following sources: mouse anti-FLAG (Sigma), mouse anti-α-tubulin (Upstate Biotech) and mouse anti-lamin A/C (Abcam). The rabbit C16 polyclonal antiserum and mouse anti-D8 mAb AB1.1 were described previously (Parkinson & Smith, 1994; Fahy *et al.*, 2008). Secondary antibodies and protein detection were performed as described previously (Bartlett *et al.*, 2002).

#### Cell fractionation.

BSC-1 cells were infected with vC4-TAP at 10 p.f.u. per cell for the indicated times and processed as described previously (Fahy *et al.*, 2008). Proteins were resolved by SDS-PAGE and detected by immunoblotting.

#### Immunofluorescence.

HeLa cells were seeded into six-well plates containing sterile coverslips (borosilicate glass; BDH) 24 h before infection at 0.5 p.f.u. per cell. At various times p.i., the cells were washed twice with ice-cold PBS and processed as described previously (Unterholzner *et al.*, 2011). Antibodies used for staining were rabbit anti-FLAG primary antibody (1 : 500 in blocking buffer; Sigma), mouse anti-p65 primary antibody (1 : 500 in blocking buffer; Santa Cruz Biotechnology), Alexa Fluor 488–donkey anti-mouse secondary antibody [1 : 500 in blocking buffer containing 10 % donkey serum (Sigma); Invitrogen] and Alexa Fluor 546–goat anti-rabbit secondary antibody (1 : 500 in blocking buffer containing 10 % donkey serum; Invitrogen).

#### Plaque-size assay.

Monolayers of BSC-1 or RK-13 cells were infected in duplicate at 50 p.f.u. per well and incubated for 72 h in medium containing 1.5 % carboxymethylcellulose. The cells were washed once with PBS and then stained with crystal violet. The diameter of 30 plaques per virus was measured using Axiovision 4.6 on a Zeiss Axiovert 200 M microscope (Zeiss).

#### Virus growth curves.

Intracellular and extracellular virus production after high or low m.o.i. was determined as described previously (Chen *et al.*, 2006) and the clarified culture supernatant was used for extracellular virus.

#### Reporter-gene assays.

Luciferase reporter-gene assays were performed by seeding HEK293T cells at a density of 10 000 cells per well before transfection with polyethyleneimine (PEI; Polysciences). Transfection reactions contained 10 ng GL3-*Renilla* control plasmid per well, 60 ng IFN-β–, AP-1– or NF-κB–firefly reporter plasmid per well (gifts from A. Bowie, Trinity College Dublin, Ireland, and G. Sen, Lerner Research Institute, Cleveland, OH, USA) and 70 ng expression vector or pcDNA4.0/TO empty vector control per well. After 24 h incubation, transfected cells were stimulated with 40 ng IL-1β or TNF-α ml^−1^ (Peprotech) for 8 h, or transfected with 800 ng poly(dA : dT) or poly(I : C) ml^−1^ (Sigma) for 24 h. The cells were harvested in Passive Lysis Buffer (Promega) and data were analysed using mars data analysis software on a FLUOstar Omega instrument (BMG Labtech). For data analysis, firefly luciferase activity values were normalized against *Renilla* luciferase activity values. Experiments were performed in biological triplicate and repeated a minimum of three times. Data are expressed as means±sd.

#### Virulence assay.

Female BALB/c mice (*n* = 5; 6–8 weeks old) were anaesthetized and infected intranasally with 5×10^3^ p.f.u. and monitored as described previously (Alcamí & Smith, 1992). Female C57BL/6 mice (*n* = 5; 6–8 weeks old) were anaesthetized and infected intradermally with 10^4^ p.f.u. and the lesion size was measured daily with a micrometer as described previously (Tscharke & Smith, 1999).

#### Analysis of cell populations.

Mice were sacrificed and the BAL fluid and lung tissue were prepared and processed as described previously (Clark *et al.*, 2006; Jacobs *et al.*, 2006). Viable cells in BAL fluid were stained with 0.4 % trypan blue (Invitrogen) and counted.

#### Statistical analysis.

Data were analysed using an unpaired Student’s *t*-test. Statistical significance is expressed as follows: *P<0.05, **P<0.005 and ***P<0.0005.
